# A DNA-Free Editing Platform for Genetic Screens in Soybean *via* CRISPR/Cas9 Ribonucleoprotein Delivery

**DOI:** 10.3389/fpls.2022.939997

**Published:** 2022-07-12

**Authors:** Saminathan Subburaj, Caroline Bedin Zanatta, Jennifer A. L. Nunn, Aline Martins Hoepers, Rubens Onofre Nodari, Sarah Zanon Agapito-Tenfen

**Affiliations:** ^1^NORCE Norwegian Research Centre AS, Department of Climate & Environment, Tromsø, Norway; ^2^Department of Crop Science, Federal University of Santa Catarina, Florianópolis, Brazil

**Keywords:** genetically modified organism, gene editing, mutagenesis, transgenesis, target deep sequencing, breeding, genetic screening, genetically modified plants

## Abstract

CRISPR/Cas9-based ribonucleoprotein (RNP)-mediated system has the property of minimizing the effects related to the unwanted introduction of vector DNA and random integration of recombinant DNA. Here, we describe a platform based on the direct delivery of Cas9 RNPs to soybean protoplasts for genetic screens in knockout gene-edited soybean lines without the transfection of DNA vectors. The platform is based on the isolation of soybean protoplasts and delivery of Cas RNP complex. To empirically test our platform, we have chosen a model gene from the soybean genetic toolbox. We have used five different guide RNA (gRNA) sequences that targeted the *constitutive pathogen response 5* (*CPR5*) gene associated with the growth of trichomes in soybean. In addition, efficient protoplast transformation, concentration, and ratio of Cas9 and gRNAs were optimized for soybean for the first time. Targeted mutagenesis insertion and deletion frequency and sequences were analyzed using both Sanger and targeted deep sequencing strategies. We were able to identify different mutation patterns within insertions and deletions (InDels) between + 5 nt and –30 bp and mutation frequency ranging from 4.2 to 18.1% in the *GmCPR5* locus. Our results showed that DNA-free delivery of Cas9 complexes to protoplasts is a useful approach to perform early-stage genetic screens and anticipated analysis of Cas9 activity in soybeans.

## Introduction

The use of the Cas protein and evolutionary components of type II bacteria were the first CRISPR-associated effectors to be widely used in genetic engineering. For this type of bacteria, defense and survival are conditions that generate cellular responses and activate protein domains (HNH, RuvC, and PI) responsible for recognizing PAM sequences in the DNA of organisms invading the bacteria (in *Streptococcus pyogenes*: NGG). Cas9 cuts the DNA strand, and the invasion is stopped (Nishimasu et al., 2014).

For gene editing *via* the CRISPR/Cas9 system, essential components, such as Cas9 endonuclease and crRNA from a sequenced genome, can be easily synthesized. The opportunity for manipulation in laboratory routines is vast, for example, in agricultural improvement where CRISPR/Cas9 makes it possible to more quickly achieve the selection, alteration, and mutagenesis of genetic traits of interest (Makarova et al., 2011; Yu et al., 2016). The system as a biotech tool works by fusing crRNA and tracrRNA (*trans-*activating crRNA) to create a single “guide RNA” with 20 base pairs that correspond to the target DNA. After the complementary sequence, Cas9 can operate by cutting double-stranded DNA (Jinek et al., 2013; Jiang and Doudna, 2017; Murovec et al., 2018; Manghwar et al., 2020).

After the double-strand break, it is natural for the ends to ligate, at this point occurs the signalization to repair by non-homologous end joining (NHEJ). This pathway can cause error-prone repair which can occur just by adding or deleting DNA nucleotides or interrupting the reading frame of a gene that could affect a protein (Hsu et al., 2013; Osakabe and Osakabe, 2015; Yu et al., 2021; Zhang et al., 2021). On the other side, using a homologous template, it is possible to direct homologous end joining – *via* homologous-directed repair pathways (HDR); in this case, the model can be used with an endogenous or exogenous template (La Russa and Qi, 2015; Wang et al., 2016).

The CRISPR/Cas9 system has often been delivered with *Agrobacterium transformation* vectors where the DNA fragment encoding the guide RNA targeting the gene of interest and the endonuclease-coding sequence are usually cloned in a transfer DNA (T-DNA) (Zhang et al., 2019; Liu et al., 2020). For targeted mutagenesis in plant cells, the strategy integrates the T-DNA in the plant genome where it constitutively expresses the CRISPR machinery (Zhang et al., 2019; Dalla Costa et al., 2020; Liu et al., 2020; Rodrigues et al., 2021). Although vectors have been widely used as a delivery tool, their application is often associated with side effects including the off-target cleavage and random insertion of foreign DNA into the genome (Amirkhanov and Stepanov, 2019). Besides that, these delivery strategies can be more exigent in techniques and equipment (Nicolia et al., 2021). Alternatively, the DNA-free RNP (preassembled Cas9-gRNA complex) delivery approach is less complex and can be delivered directly into living cells. In plants, the removal of the cell wall (cellulose, polysaccharides, hemicellulases, and pectins) with enzymatic treatment possibilities the CRISPR delivery by polyethylene glycol (PEG) (Metje-Sprink et al., 2019). This approach was demonstrated as efficient in protoplasts of several plant species including *Arabidopsis thaliana*, rice (*Oryza sativa*), lettuce (*Lactuca sativa*) (Woo et al., 2015), tobacco (*Nicotiana tabacum and N. attenuata*), petunia (*Petunia* × *hybrida*) (Subburaj et al., 2016; Yu et al., 2021), corn (*Zea mays*) (Sant’Ana et al., 2020), grapevine (*Vitis vinifera*), apple (*Malus* × *domestica*) (Malnoy et al., 2016), wheat (*Triticum aestivum*) (Liang et al., 2017), tomato (lat. *Solanum lycopersicum*) (Nicolia et al., 2021), cabbage (*Brassica oleracea*), Chinese cabbage (*Brassica rapa*; Murovec et al., 2018), banana (*Musa spp*.) (Wu et al., 2020), and pepper (*Capsicum annum*) (Kim et al., 2020).

In soybean, the most recent studies were related, which described an optimized PEG-calcium-mediated transformation method to do transient gene expression into soybean protoplasts (Wu and Hanzawa, 2018). The genome edition uses preassembled binary vectors with CRISPR/Cas9 delivered by PEG into protoplasts (Sun et al., 2015), and the use of type V of CRISPR with Cpf1 endonucleases proteins (LbCpf1 and AsCpf1) and crRNAs preassembly delivered into the protoplast by PEG (Kim et al., 2017, 2020). The *CPR5* gene (Glyma_06g14800) that regulates the growth of trichomes was also used for knockout *via* the CRISPR/Cas9 system successfully, eliminating the gene using somatic embryos transformed by a plasmid with gRNA and preassembled *Staphylococcus pyogenes* (Spy) Cas9 incorporated into a vector for biolistic transformation (Campbell et al., 2019).

To contribute to genome editing approaches in soybean, we have developed a DNA-free friendly plant genome editing platform where the CRISPR/Cas9 system and PEG are delivered in protoplasts. We use the knowledge since available to target Glyma_06g145800 a single copy gene, without repetitive genome sequence, involved in cell division and endoreduplication in trichomes. The results of targeted deep and Sanger sequencing analysis showed an InDel mutagenesis efficiency of 4.2 to 18.1% for the targeted distinct sites of endogenous *Glycine max CPR5* locus (*GmCPR5*). This approach is useful for generating mutant cell lines in protoplasts without the possibility of backbone integration and for investigations into the complexity and interactions between cellular physiological responses to gene editing by CRISPR/Cas9.

## Materials and Methods

### Platform Development

**FIGURE 1:**
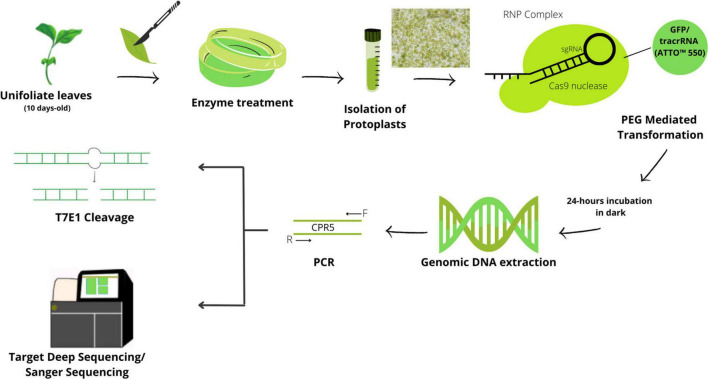
Schematic protocol for CRISPR/Cas9-mediated DNA-free/transient genome editing in the soybean. The protoplasts were isolated from young seedlings of unifoliate leaf strips with enzyme solution. Isolated protoplasts were transformed with preassembled CRISPR-RNP complexes *via the* PEG-mediated method. The transformed protoplasts were subjected to either genomic DNA extraction or fluorescence-activated cell sorting (FACS) of Cas9-GFP expressing protoplasts for an enrichment followed by DNA extraction or to cultivation. Target sites are amplified by PCR followed by T7E1 validation of mutation and high-throughput sequencing for estimation of mutation efficiency.

This paper describes a platform for genetic screens in gene-edited soybean lines. The focus of the platform is the development of knockout gene-edited cell lines without the transfection of DNA-based vectors. To enable genomic screens and investigations, the platform consists of delivering CRISPR as RNP reagents to protoplasts with the aid of PEG solutions (Figure 1). In addition to establishing a pipeline for editing genes in soybean, the platform also works to improve the effectiveness of CRISPR techniques, enhance delivery methods, and create infrastructure and resources to enable their use on large- and small-scale assays. Importantly, the platform can also be expanded to include other species, other types of molecular profiling, and other CRISPR-delivery methodologies. The platform was developed by two different laboratories which served as a validation for the protocols applied.

### Plant Material and Growth Conditions

Seeds of non-transgenic soybean varieties (*Glycine max*) were sown and grown in soil (composed of peat, vermiculite, organic waste, and limestone) for 10 days in a growth chamber (Enviro Plant^®^). Growing conditions were standardized to 60% humidity, 25°C temperature, and photoperiod (10/14-h light/dark cycle). Plants were subjected to regular sunlight at a photosynthetic flux of 625 Umol/m^2^/s^–1^ and photosynthetic active radiation (PAR) of 1,200 lumens/m^2^. Unifoliolate green leaves were used for protoplast isolation.

### Selection of Target Region and Guide RNA Design

**FIGURE 2:**
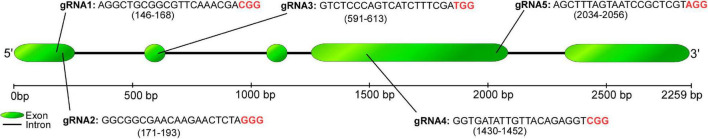
Schematic representation of the *GmCPR5* locus and design of gRNAs. The location of target sites was shown by engineered gRNAs 1, 2, 3, 4, and 5 along with their sequences. The PAM motifs are indicated in red.

*CPR5* gene (Glyma_06g15080; gene ID: 100791857) was selected as a model gene in this study. The *CPR5* gene has only a single copy in the soybean genome (NCBI GenBank Glycine_max_v4.0) and a phenotype associated with the growth of trichomes (Campbell et al., 2019). According to available sequence information of *CPR5* in the NCBI and SoyBase databases^1^, a partial *CPR5* locus flanking the targeted site was amplified and sequenced by the Sanger platform (ABI3500xl Applied Biosystems) with identical matching results from the *Glycine max* CPR5 NCBI sequence. In order to design the gRNAs from the gene, four online prediction software, namely CRISPOR (Concordet and Haeussler, 2018), CRISPR RGEN Tools (Bae et al., 2014; Park et al., 2015), CRISPRdirect (Naito et al., 2015), and CHOPCHOP v3 (Labun et al., 2019), were used. Three different target sites corresponding to the exons 1, 2, and 4 of *CPR5* locus were chosen which ranked as top among the potential five gRNA targets in most software (Figure 2). For the selection of efficient gRNAs, predicted cleavage efficiency score, high GC% (content between 40 and 70%), high out-of-frame scores or frameshift rate (complete knockout efficiency on-target), and minimum number of mismatches at off-target sites were taken into consideration. The list of designed gRNAs is shown in Supplementary Table 1. *In vitro* transcription of sgRNAs and synthesis were carried out using the HiScribe™ Quick T7 High Yield RNA Synthesis Kit (New England Biolabs, Ipswich, MA, United States) according to the manufacturer’s instructions.

### *In vitro* Cleavage Assay

Cas9 RNP activity was determined *in vitro* using 20 μl reactions containing tracrRNA [1 μg/μl], each crRNA [1 μg/μl], and 1.35 μl of duplex buffer for preassembly at 95°C for 5 min. Cas9 [10 μg/μg] and NEB 3 buffer (1×) (Integrated DNA Technologies, Coralville, IA, United States) were then added and incubated at 25°C for 15 min. The reaction was stopped with 0.5 μl of proteinase K (8,000 μ/μl). The target site containing 300 ng of PCR product was amplified (Platinum PCR SuperMix High Fidelity, Invitrogen) using the following cycling conditions: 2 min at 94°C, followed by 30 cycles of 30 s at 94°C, and 1 min at 68°C, and then visualized by running a 2% agarose gel and staining with GelRed^®^ for UV imaging. Primers to amplify the target DNA region were designed by the PrimerQuest tool (Integrated DNA Technologies Inc., Skokie, IL, United States), and their annealing temperature and amplicon sizes are available in Supplementary files (Supplementary Table 2).

### Protoplast Isolation

Protoplasts were isolated from 10-day-old grown seedlings. After removing their midribs, 10–12 young “unexpanded” unifoliate leaves were sliced transversely into 0.2–0.4 mm thick slices and placed into 20 ml of enzyme solution containing Viscozyme^®^ L (0.8%) + Celluclast^®^ 1.5L (0.4%) + Pectinex^®^ Ultra SPL (0.4%) mixtures (Sigma-Aldrich, Germany), MES (5 mM), and mannitol (9%) in cell and protoplast washing (CPW) salts (Frearson et al., 1973) at pH 5.8. The enzyme digestion was carried out in a gyratory shaker at 60 rpm for 2, 4, 6, and 8 h in dark conditions. After enzymatic digestion of cell walls, the solution was filtered through a 0.45-μm nylon mesh and the filtrate was further diluted with 10 ml of CPW salts with 9% mannitol (9M CPW) to stop the enzyme reaction. Then, the protoplast cells were pelleted by centrifugation at 100 × *g* for 5 min, and harvested cells were further washed three times by resuspending them with a 10 ml of 9M CPW, followed by a centrifugation at 100 *g* for 5 min. Washed cells were resuspended with 1–2 ml of 9M CPW, and 10 μl of this suspension was loaded into the Neubauer cell chamber for calculating the number of protoplasts under a light microscope. The resuspended cells (1–2 ml) were further dispersed in 9–8 ml of 9M CPW solution and rested at 4°C for 1 h before the viability counting and the PEG transfection were conducted. The viability of protoplasts was determined according to a previous study (Adedeji et al., 2020); briefly, a 100 μl of cells was incubated with 2 μl of 0.5% of fluorescein diacetate (FDA) (Sigma-Aldrich, Germany) at 25°C for 5 min and observed under a fluorescence microscope.

### Protoplast Transfection With Cas9 Ribonucleoprotein (RNP)

Each transfection experiment consisted of 1 × 10^6^ protoplasts in MMG solution (4 mM MES, 0.4 M Mannitol, and 15 mM MgCl). RNP complexes were composed of 30 μ g gRNA [1 μg/μl] and 10 μ g Cas9 [10 μg/μl] molecules at a 3:1 molar ratio. The reagents were purchased from Integrated DNA Technologies (IDT) (California, United States) and Sigma-Aldrich (Darmstadt, Germany), and the complexes were mixed, following the manufacturer’s instructions. The complex was then mixed with protoplast suspensions followed by an equal volume of freshly prepared PEG solution (40% PEG-4000, 0.4 M mannitol, and 0.1 M CaNO_3_) which was added and mixed by gentle shaking. Cells were incubated at room temperature in the dark for 20 min. The transfection was induced at 25°C for 23 min in darkness and stopped by a gradual dilution and gentle mixing of the reaction content with ascending series of 9M CPW (0.6, 1.2, 2.4, and 4.8 ml) over a 30-s period at 2-min intervals. Transfected protoplasts were centrifuged at 100 × *g* for 7 min, and the pellet was retained. Then, the protoplast pellets were resuspended in 1 ml of KP8 liquid medium (Kao, 1977) containing 3% sucrose and 9% mannitol at pH of 5.8 and incubated at 25°C in darkness for 16–24 h prior to DNA extraction. For microscopic fluorescent analysis, protoplasts were transfected with either GPF-tagged Cas9 (Sigma-Aldrich, Germany) or fluorescently labeled tracrRNA–ATTO™ 550 (IDT, United States).

### Mutation Detection and Sequencing

Genomic DNA was extracted from protoplast transfectants after 24 h using Plant DNAzol™ Reagent (Invitrogen Co., Carlsbad, CA, United States) according to the manufacturer’s instructions. CRISPR-targeted sites in *GmCPR5* loci were amplified from genomic DNA using designed primers as described *in vitro* cleavage assay. Amplified PCR products were subjected to T7 endonuclease I (T7E1) assay after denaturation and re-anneal process. The T7EI assay was performed as per the manufacturer’s instructions for the Alt-R^®^ Genome Editing Detection Kit (Integrated DNA Technologies, Coralville, IA, United States). T7E1-digested PCR products were resolved on a 2% agarose gel. PCR products were further analyzed by targeted deep sequencing using the Illumina NovaSeq™ 6000 platform at Novogene Europe (Cambridge Science Park, United Kingdom). Mutation patterns at cleavage sites were analyzed by the Cas-Analyzer program in CRISPR RGEN Tools^2^ and calculated according to the previous study (Subburaj et al., 2016). Sanger sequencing was performed with purified PCR product (1 ng/μl) using ExoSAP-IT Express (Thermo Fisher Scientific) following the manufacturer’s instructions: forward primer (3.2 μM), 1 μl of BigDye Terminator v3, 4 μl sequencing buffer, and 13 μl water. Sanger was performed (3500×, Genetic Analyzer, Applied Biosystems, São Paulo, Brazil). To determine and characterize the types of insertions and deletions (InDels) at the target location, DECODR (Bloh et al., 2021) was used. Protoplasts edited with CRISPR/Cas9 were compared to their negative controls and also sequenced.

### Microscopic Analyses

Bright-field images of isolated protoplasts were captured using phase contrast on a Zeiss Primovert compact inverted microscope. Fluorescence images were obtained with the confocal laser scanning microscope Zeiss LSM800 using a 488-nm diode laser for green fluorescent protein (GFP)-labeled and FDA-stained cells. For transmitted light detection images, the electronically switchable illumination and detection module (ESID) was used.

## Results

### Protoplast Isolation and Transfection

**FIGURE 3:**
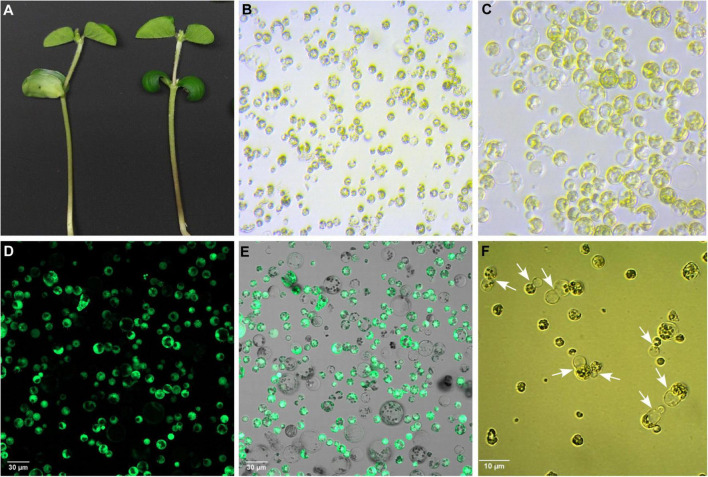
Isolation, purification, and cultivation of protoplasts from *Glycine max* cv. OAC Bayfield. **(A)** Unifoliate leaves of 10-day-old soybean seedlings. **(B)** Freshly isolated protoplasts. **(C)** Washed and purified protoplasts. **(D)** FDA-stained protoplasts subjected to confocal fluorescence microscopy to visualize viable cells (GFP-positive). **(E)** Merged image of green channel (GFP) and ESID channel (transmitted light detection) showing all protoplasts. **(F)** Protoplasts undergoing cell division (indicated by white arrows) in culture medium after 3 days of isolation.

**FIGURE 4:**
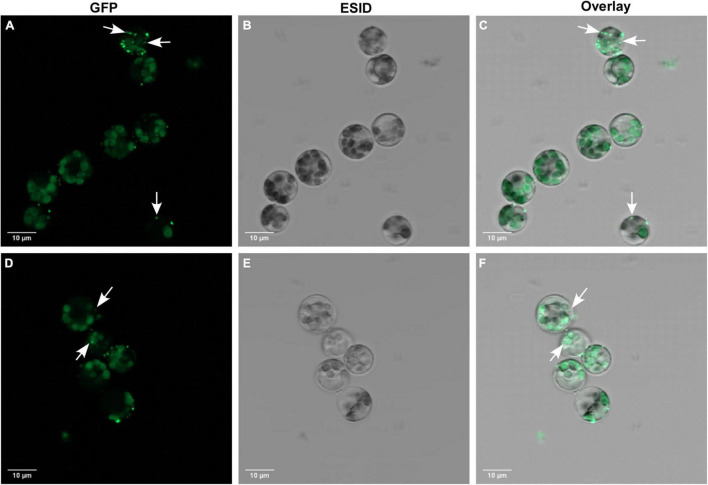
Cellular localization of GFP-Cas9 RNP complexes in transfected protoplasts from *Glycine max* cv Bayfield. **(A,D)** Confocal fluorescence microscopy showing GFP-Cas9 located inside transfected protoplasts using the green channel. White arrows indicate internalized localization of GFP-Cas9. **(B,E)** The same protoplasts as depicted in **(A,D)** showing bright-field images using the ESID channel. **(C,F)** Are overlay images of green and ESID channels. White arrows indicate internalized localization of GFP-Cas9.

In this study, we used 10-day-old unifoliate leaves for protoplast isolation and further cell editing (Figures 3A–F). By using the 1 × VCP enzyme solution, we obtained a maximum yield of 1.5 × 10^6^ cells after 4 h of incubation, in which 77% of cells were alive during the viability test using FDA. The results obtained suggest optimized conditions for fast isolation of protoplast cells that can be used for the study of knocked out gene regulation. The visualization of RNP complex internalization into cells was verified using ATTO-labeled TracrRNA and GFP-labeled Cas9 (Figures 4A–F). Internalization efficiency was calculated with an unsupervised eye to approximately 38% after 18 h of incubation.

### *In vitro* Cleavage of Soybean Genomic Target Sites

**FIGURE 5:**
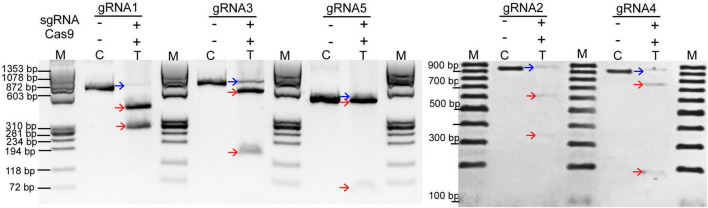
*In vitro* cleavage assay. The *in vitro* transcribed or purchased sgRNAs at *GmCPR5 loci* were mixed with SpCas9 and PCR templates of target sites (gRNA1–5) for *in vitro* digestion and resolved on 2% agarose gel. Lanes M, DNA ladders; C, PCR wild type (control untreated); T, treated with sgRNAs and SpCas9. The parental and cleaved fragments are indicated with blue and red arrows, respectively.

Three partial genomic regions in the *CPR5* loci with flanking exons 1, 2, and 4 were analyzed and confirmed by Sanger sequencing (NCBI accessions: OK631878, OK631879, and OK631880), and subsequently, five sgRNAs were designed. Each of the sgRNAs is 20 nucleotides in length, and they pair with their corresponding 20 nucleotides at target sites in *GmCPR5* locus to aid CRISPR/Cas9 system to make site-specific DSBs. To determine the specificity of the CRISPR-RNP complexes (recombinant Cas9 + *in vitro* transcribed gRNAs), *in vitro* cleavage assay was performed. The 657 bp PCR amplicon of *GmCPR5* for gRNA1 was cleaved into ∼ 397and ∼ 260 bp as expected. For gRNA2, digestion of 931 bp PCR product generated two fragments of ∼ 600 and ∼ 331 bp. Likewise, cleaved fragments of 522 and 138 bp in gRNA3 (660 bp), 763 and 190 bp in gRNA4 (953 bp), and 439 and 47 bp in gRNA5 (486 bp) were noted in the cleavage assay (Figure 5 and Supplementary Table 2). Our results show that the designed sgRNAs were able to efficiently cleave at their corresponding target regions of *CPR5* (Figure 5).

### Targeted Mutagenesis of Soybean Using CRISPR/Cas9 Ribonucleoproteins

**FIGURE 6:**
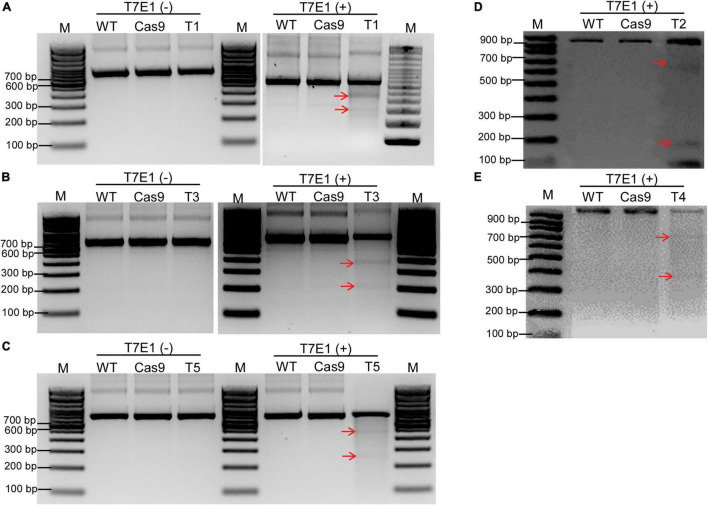
Detection of site-directed mutagenesis at target sites on *GmCPR5 loci* using direct delivery of RGEN RNPs. **(A–E)** T7E1 digestion resulting gel images for the transformants of T1–T5. Lanes M, DNA ladder; WT, untransformed wild type (control) to each target site; Cas9, transformed with SpCas9 only; T1–T5, transformed with RNPs; T3T7E1(–), negative control (undigested). Cleaved fragments are indicated with red arrows. Images 6E and 6F have been processed for better visualization of bands. This process is in accordance with Frontiers Policies and Publication Ethics Guidelines.

Ribonucleoprotein complexes (Cas9 protein + gRNA) were transfected into the soybean protoplasts using the above-mentioned protoplast transformation system to make site-directed mutations in *GmCPR5*. After 24 h of transfection, the genomic DNA was extracted from control and transfectant protoplasts for mutation T7E1 assay. T7E1 digestion assay showed the appearance of cleaved DNA products for all the sgRNA-transfected samples (Figure 6). This confirmed that there were induced InDel mutations at the corresponding targeted sites within the *GmCPR5* locus, whereas in the negative controls, WT and Cas9, no cleavages were detected.

### Mutation Range Characterization in Soybean Edited Cell Lines

In order to calculate the mutation frequency and characterize the site-targeted mutation patterns in the *GmCPR5* gene locus, we performed the analysis of sequencing results obtained from targeted deep sequencing (for gRNAs 1, 3, and 5) and also Sanger sequencing (for gRNAs 2 and 4) for the genomic DNA from targeted protoplast transformants. The designed nested PCR primers and their corresponding amplicons from control and protoplast transformants are provided in supplementary files (Supplementary Table 2 and Supplementary Figure 1). The obtained raw sequencing data for RNP transformants of T1, T3, and T5 are available under accession number PRJNA785774 at the National Center for Biotechnology Information (NCBI) BioProject. The targeted deep sequencing results showed that the T1, T3, and T5 RNP transformants were found to possess a range of mutations, such as InDels, in their corresponding target site, whereas no significant mutations were observed in wild-type, negative control samples (Table 1). Some of the RNPs produced an equal proportion of insertions and deletions (T1) or either only insertions (T3) or deletions (T5) with higher frequencies. Based on the number of insertions and deletions for each RNPs, the calculated ratio of deletion to insertion was found as about 49.2:50.8 in the three target sites. Furthermore, by analyzing the total number of mutated sequences in contrast to the total number of obtained reads, the results showed that the three different gRNAs (gRNA1, 3, and 5) generated mutation frequencies ranging from 5.5 to 18.1% with an average mutation frequency of 12.9 ± 3.1% in the *GmCPR5* locus.

**TABLE 1:** Estimation of mutation rate in *GmCPR5* gene sequences in wild-type non-transformed and transformed protoplasts by targeted deep sequencing in soybean protoplasts using direct delivery of RNP’s.

Protoplast samples	Wild-type negative control				
	Total	Indel	Indel frequency (%)	Insertion^a^	Deletion^b^
Negative T1	4,588,627	189	0.004	15	174
Negative T3	2,510,574	160	0.006	24	136
Negative T5	3,386,910	203	0.005	30	173
**Average^c^**	3,495,370 ± 491,799	184 ± 10.3	0.005 ± 0.0005	23 ± 3.6	161 ± 10.2

	**Cas9 RNP transformants**				

T1	5,313,317	961,257	18.1	542,715	418,542
T3	5,752,838	316,104	5.5	263,935	52,169
T5	5,690,884	863,729	15.2	280,150	583,579
**Average^c^**	5,585,680 ± 112,146	713,697 ± 163,936	12.9 ± 3.1	362,267 ± 73766.8	351,430 ± 128,216

*^a^Number of insertions was analyzed. ^b^Number of deletions was analyzed. ^c^Values of average and standard deviation error.*

**FIGURE 7:**
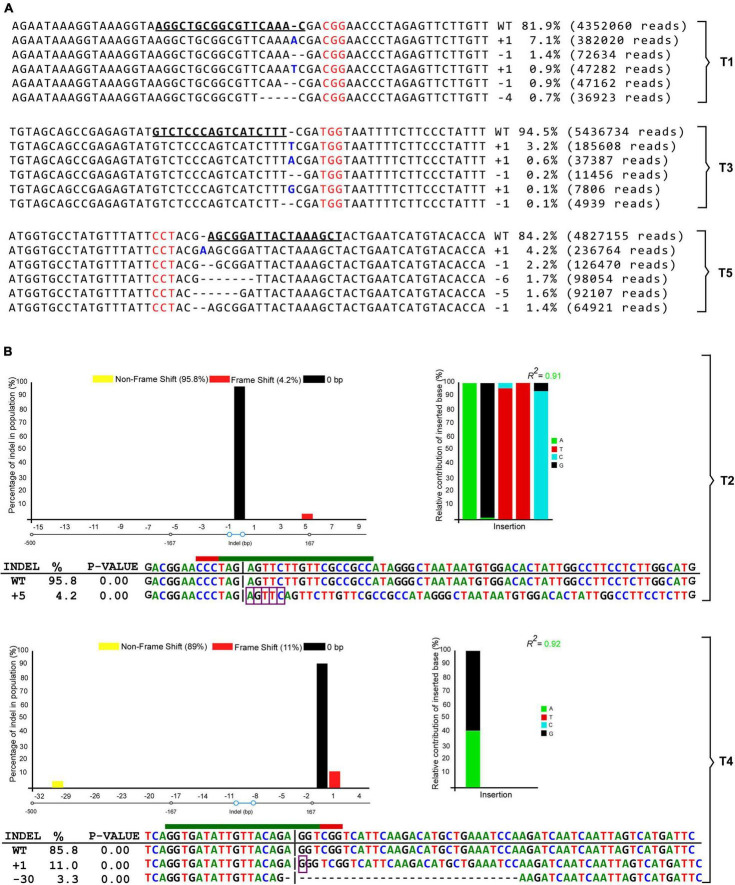
Mutation patterns observed by targeted deep and Sanger sequencing for the corresponding target sites at *GmCPR5* loci are shown. **(A)** Distribution of the five most frequent alleles along with their mutation pattern, contribution percentage, and read count observed with the Cas-Analyzer around targeted sites in *GmCPR5* for T1, T3, and T5. Wild-type (WT) nuclease target sequences were in bold and underlined. PAM sites are denoted by the red font. Insertions and deletions are shown in bold font (blue) and horizontal dashed lines, respectively. **(B)** The result of editing efficiency and mutation patterns analyzed with DECODR software for T2 and T4. The top panels display the graphs for the InDel distribution rate. The bottom panel shows the list of deconvoluted InDel-containing sequences as alignments along with InDel types and percentages (%). Insertion (highlighted with purple rectangles) and deletion (marked with horizontal dashed lines) of mutations are shown in alignments. A 20 bp target and 3 bp PAM site are depicted with green and red lines, respectively.

The targeted deep sequencing results from T1, T3, and T5 transformants showed a range of mutational profiles of which we selected five. The five most contributing mutation sequencing patterns (highest frequencies) are presented in Figure 7A. These five most frequent alleles themselves contributed 11, 4.2, and 11.1% of the total mutation rates for T1, T3, and T5, respectively (Figure 7A and Table 1). It was observed that the targeted sites were mutated with InDels ranging from + 1 to –6 nt in length, in which T5 produced a maximum of –6 bp deletion. Interestingly, we have the insertion of an adenine as one of the most frequent outcomes in gRNAs 1 (7.1% with 382020 reads) and 5 (4.2% with 236764 reads) analyzed by target deep sequencing (Figure 7A). In addition, all mutant *GmCPR5* alleles derived from T1, T3, and T5 were compared with wild-type *GmCPR5* for further characterization. The results indicated that these mutations were frameshift types, which would produce in-frame premature stop codons at mRNA and cause loss of function in *GmCPR5* alleles.

To assess the CRISPR-induced mutations at targeted sites of T2 and T4 transformants, we have PCR-amplified and Sanger-sequenced each of the targeted sites. The obtained chromatogram files from Sanger sequencing are provided in Supplementary File 1. The corresponding sequencing chromatograms were analyzed by DECODR online CRISPR analysis software. Our Sanger sequencing results showed that RNP transformants of T2 and T4 samples had an InDel rate of 4.2 and 14.3%, respectively (Figure 7B). Interestingly, the sequencing analysis of T2 showed the highest contributing mutational profile of + 5 bp (AGTTC) insertion (4.2%) at the targeted site (Figure 6B), whereas the T4 had the highest contributing sequence variant of + 1 bp (G) insertion (11%) followed by a −30 bp (GGTCGGTCATTCAAGACATGCTGAAATCC) deletion (3.3%) sequence contribution. Upon characterizing the mutant alleles, + 5 bp (T2) and + 1 bp (T4) were identified as frameshift types that would affect the reading frame and cause complete loss of functions in *GmCPR5*, whereas −30 bp (T4) were found to be a mutant with in-frame deletion in the target site which could alter or partially eliminate protein function of *GmCPR5* (Figure 7B).

Overall, the results of both targeted deep and Sanger sequencing analysis demonstrate that the five different targeted distinct sites (T1–5) had mutation frequencies ranging from 4.2 to 18.1% in the *GmCPR5* locus (Table 1 and Figure 7). Among all the RNP-induced mutations, + 1 insertions at the fourth nucleotide upstream of the PAM sites were prevalent and most frequently observed in all the targeted sites except for T2 (Figure 7).

## Discussion

### DNA-Free Platform Performance in Soybean

Soybean methods, already known, have been used for the edition by CRISPR including the construction of binary vectors with the insertion of T-DNA (Chen et al., 2021), biolistic bombardment (Sun et al., 2015), and electroporation treatments (Liu et al., 2019a). Here, we report for the first time that CRISPR/Cas9 was used in the DNA-free target mutagenesis of soybean material by RNP delivery into protoplasts without the application of any DNA vectors (Figures 1–6). The results obtained from targeted deep and Sanger sequencing analysis revealed that at five target sites (T1-5), we successfully mutated the *GmCPR5* locus at frequencies ranging from 4.2 to 18.1% (Table 1 and Figure 7). The mutation frequency observed in our study is similar to frequencies observed by other DNA-containing gene-edited platforms for soybean. The vector *Agrobacterium tumefaciens* carrying CRISPR/Cas9 and gRNAs was utilized to knockout the male-sterile gene (*GmAMS1*) in soybean cotyledons; in this case, the mutation frequency was 25% (Chen et al., 2021). In another study, the same vector, with gRNA/Cas9, was applied to get knockout the soybean flowering gene (*GmFT2a*), and the mutations obtained were between 12.1 and 15.8% (Cai et al., 2018). Knockout obtained in genes related to fatty acid (*FAD2-2 omega-6*) by CRISPR/Cas9-mediated in the binary vector *Agrobacterium tumefaciens* produced 21% maximum mutation efficiency (Amin et al., 2019). Using the CRISPR-Cas9 system in the *Agrobacterium rhizogenes* mediated into soybean protoplasts using PEG, the mutation frequencies obtained in the three gene targets (Glyma06g14180, Glyma08g02290, and Glyma12g37050) ranged from 14.7 to 20.2% (Sun et al., 2015). The Agrobacterium transformation targeting the glucosyltransferase soybean gene (Glyma07g14530) achieved a maximum mutation efficiency of 21%, while in events in which transformation was performed by biolistic, the frequency was only 12.5% (Jacobs et al., 2015). Our review of the literature showed that gene editing in soybean, regardless of the techniques applied, did not reach efficiency greater than 25% and these were quite variable depending on the target gene sequence, the sgRNA sequence, and material/tissue type (Tsai et al., 2015; Zischewski et al., 2017).

Despite the common understanding that NHEJ mechanisms will trigger insertions and deletions 3 bp after PAM NGG sites (Shrivastav et al., 2008), we showed different patterns which do not seem to follow that rule. The NHEJ repair after DNA cleavage produced different mutation patterns, and it was observed that the target sites were mutated with InDels ranging from + 1 to –7 nt in length. We also observed big deletions outside the NHEJ repair. The screening in the sequence made by Sanger sequencing revealed that the gRNA 5 produced a deletion with –30 bp (3.3%) (Figure 7B). Other studies also observed the deletion of larger sequences when samples were analyzed using Sanger sequencing approaches. A range of deletion sizes from –7 to –77 bp in two different cultivars (DT26 and Maverick) was detected after the knockout of three *GmGOLS* genes (*GmLox1*, *GmLox2*, and *GmLox3*). The seeds obtained after knockout showed significant shifts in stachyose, raffinose, verbascose, and sucrose (Do and Chu, 2020). Using Agrobacterium strains to edit and get knockout of lipoxygenase-free soybean genes, different mutation patterns ranging from + 1 to –8 nt were found in PCR products sequenced by Sanger (Wang et al., 2020).

A knockout using the CRISPR system in Agrobacterium-mediated gene to flowering-related genes (E1) in soybean produced two different deletion patterns. The mutations identified by sequence peaks showed that homozygous mutations at the target sites were 11 bp deletion and 40 bp deletion. The authors related that this mutation frameshift resulted in premature translation termination codons of 79 amino acids (aa) and 88 aa, respectively (Han et al., 2019). The CRISPR/Cas9 system was used to gene edit the target gene (*GmJAG1*) predicted to modulate yield in the low-latitude was applied in soybean. Although the authors do not mention the delivery method, the sequencing results obtained by Sanger showed that in the T1 segregation population, there was deletion (−4 bp) and lost start codons (Cai et al., 2021). Interestingly, our gRNA2 results diverge from a previous study that first designed this gRNA sequence. Campbell et al. (2019), using an expression vector and biolistic transformation, obtained four different allelic combinations that ranged from –2 to –21 bp of deletion using gRNA2 (Campbell et al., 2019). In our study, using the same gRNA sequence, we did not obtain any series with deletion; on the contrary, we have obtained an insertion of + 5 bp (AGTTC) at the target site. These results can demonstrate that the differences found may be related to the genotype used, tissue issue, delivery method, or even the analytical methods such as the sequencing platform and the software used for the deconvolution of Sanger sequencing data. Overall, in this study, we demonstrated that our editing platform is (1) effective for in-frame mutations in the trichome gene; (2) satisfactory mutation efficiency which is suitable for gene expression studies; and (3) advantageous toward the absence of external DNA backbone integration into the genome.

### DNA-Free Gene Editing in Other Species

RNP-mediated genome engineering has been demonstrated in protoplasts for various model plants and crop species including Arabidopsis, tobacco, rice (Woo et al., 2015), Petunia (Subburaj et al., 2016; Yu et al., 2021), apple, grape (Malnoy et al., 2016), maize (Sant’Ana et al., 2020), potato (González et al., 2020), cabbage (Murovec et al., 2018), chickpea (Badhan et al., 2021), and soybean (Kim et al., 2017; Kim and Choi, 2021).

Direct delivery of DNA-free recombinant Cas9 proteins was found to be as efficient or even more efficient in some cases compared to other techniques, and it also limits the unwanted off-target mutations. Synthesis of unique sgRNAs, prediction of unique target sites, and the molar ratio of Cas9:sgRNA would also facilitate the on-target specificity of CRISPR/Cas9. In order to achieve a higher mutation rate in endogenous *CPR5* in the soybean protoplast system, we have tried a fixed Cas9 ratio along with several different sgRNA ratios (1:1, 1:2, and 1:3) in a similar way to previous studies (Woo et al., 2015; Subburaj et al., 2016). We found that the only ratio of 1: 3 (10 μg Cas9 + 30 μg sgRNA) was very suitable, in which we confirmed the induced mutations during T7E1 analysis (Figure 6), while other ratios showed only negative results. This suggests that adjustment of the molar ratio of Cas9 to sgRNA would be a crucial factor to achieve a higher mutation rate of interest genes as noted in recent studies (Malnoy et al., 2016; Murovec et al., 2018).

In this study, with a fixed Cas9 to sgRNA ratio (1:3), the mutation rates of 4.2–18.1% were noted from sequencing results including target deep and Sanger assays in soybean protoplasts within 24 h of transfection (Table 1 and Figure 7). The editing efficiency in this study corresponded well with many previous reports. The editing efficiency has been observed to greatly vary in plant protoplasts using the RNP-mediated CRISPR/Cas9 system. Editing efficiencies could likely be attributed to explant, species, sgRNA efficiency, Cas protein activity, transformation, and detection methods. Using PEG-mediated protoplast assays, editing frequencies of 16, 44, and 19% have been reported in Arabidopsis, tobacco, and rice, respectively (Woo et al., 2015). In garden petunia, 5.30–17.83% (*Nitrate reductase*) and 9.99–26.72% (*flavone 3’ hydroxylase*) of editing frequencies were obtained (Subburaj et al., 2016; Yu et al., 2021). In cabbage species, a minimum of 0.09% (*Brassica oleracea* var. *capitata f. alba*) and a maximum of 24.51% (*Brassica rapa* subsp. *pekinensis*) have been observed (Murovec et al., 2018). Likewise, mutation frequencies of 0.5–6.9% in apple, 0.1% in grapes (Malnoy et al., 2016), 0.19–0.92% in cavendish banana (Wu et al., 2020), 0.5–11.3% in sweet pepper (Kim et al., 2020), and 0.85–5.85% in maize (Sant’Ana et al., 2020) have been reported in protoplasts.

LbCas12a-RNP (previously named Cpf1) has been considered an alternative approach to the SpCas9-RNP system (Swarts and Jinek, 2018) because of its smaller protein size and induction of large deletions. In this study, using the SpCas9-RNP system, we obtained the highest mutation frequency of 18.1% in soybean leaf mesophyll protoplast for T1 RNP (Table 1). It is significantly higher than the previously reported mutation frequency of 11.7% for *FAD2-1A* in soybean leaf mesophyll protoplast by the cpf1-RNP system (Kim et al., 2017). Furthermore, characterizing the mutation patterns, the designed five sgRNAs (T1–T5) were successfully induced the InDels at target sites, which are 1–7 (target deep) and 1–30 bp long (Sanger sequencing) that would change the open reading frame of *GmCPR5* and cause loss of their function. These observed mutation patterns and their sizes corresponded well with the previous genome editing studies using CRISPR/Cas9 (Woo et al., 2015; Subburaj et al., 2016; Sant’Ana et al., 2020). These results demonstrate that the direct DNA-free delivery of CRISPR RNPs to soybean protoplast is improvised in this study which could produce mutations on targeted distinct sites of endogenous target genes through DSBs. Currently, we developed and optimized an efficient genome editing platform in soybean; in addition, future studies on the regeneration of whole plantlets from CRISPR/Cas9-edited protoplast cells will facilitate the development of DNA-free genome editing of soybean and its related legume crops.

### DNA-Free Editing as a Tool for Genetic Screening in Plants

In this study, we demonstrated a DNA-free genome editing approach to edit the endogenous *GmCPR5* locus using CRISPR/Cas9-based technology. We used RNP-mediated CRISPR/Cas9 system as a safe and effective tool to make site-directed mutations. The Agrobacterium-mediated and particle bombardment-mediated transformation methods are also commonly used to deliver the plasmid DNA carrying CRISPR (Cas9 and sgRNA) reagents into plant tissues and cells. However, these methods are limited by their unwanted off-target mutations, caused by random integration of CRISPR expression cassettes into genomes and followed by genome damage (Banakar et al., 2019; Liu et al., 2019b). Furthermore, the continuous expression of integrated transgene cassettes could have resulted in continuous damaging of genomic DNA which leads to off-target mutations (Hashimoto et al., 2016). The random integration of genome editing components into the recipient genome would also be considered as genetically modified organism (GMO) and raise concerns among enforcement institutions (Zhang et al., 2015). The plasmid-mediated transformation of CRISPR/Cas9 into living cells often needs optimized compatibility of promoters and terminators in the expression system. In some cases, the DNA-based expression of Cas9 protein has been found toxic to living cells (Morgens et al., 2017; Foster et al., 2018). To overcome the above-mentioned drawbacks, transiently expressed plasmid DNA carrying Cas9 nucleases along with sgRNA(s) has been successfully delivered into plant cells (Zhang et al., 2016). Alternatively, the direct delivery of DNA-free proteins, such as the RNP complex (preassembled Cas9 protein and sgRNA), could also be delivered into living cells. Using RNPs has been found to decrease off-target effects as it could be easily degraded by cell endogenous proteases and nucleases (Kim et al., 2014). Organisms edited by RNPs also are not restricted by GMO rules as it involves using recombinant DNA (Araki and Ishii, 2015; Wolt et al., 2016). Recent CRISPR/Cas9 studies on soybean have successfully produced trait-specific knockout lines using Agrobacterium-mediated transformation methods which could be considered GMO (Han et al., 2019; Wang et al., 2020).

CRISPR/Cas9-based RNP-mediated system has been more effective in terms of preparation, delivery, screening of CRISPR components, and generating target-specific mutations at the targeted locus to produce transgene-free engineered plants (Subburaj et al., 2016; Yu et al., 2021). By using RNPs, site-directed mutations in plants could easily be genotyped, suggesting that they are sensitive and easy to approach (Liang et al., 2018). Most studies have exploited the NHEJ-mediated genome editing using RNP-mediated CRISPR/Cas9 system, which usually creates imprecise small InDels. However, in maize, RNPs were used to make site-directed mutations through the HDR pathway by introducing donor DNA templates (Svitashev et al., 2016). A study in Arabidopsis reported a 223 bp deletion using RNPs (Woo et al., 2015), indicating that metabolic engineering of plants is feasible in the future by inducing large deletions in the genome. RNP-mediated editing relies on the perfect delivery method, compared with various RNP delivery methods such as electroporation (Lee et al., 2020), lipofection (Liu et al., 2020), and particle bombardment (Svitashev et al., 2016; Liang et al., 2017; Banakar et al., 2020). PEG-mediated transfection was noted as a predominantly used method as it has also been successfully demonstrated for various model plants and crop species (Woo et al., 2015; Subburaj et al., 2016; Kim et al., 2017; Sant’Ana et al., 2020; Yu et al., 2021). The PEG-mediated method could be more efficient, cost-effective, and simple in terms of delivering RNPs into plant cells than other methods like particle bombardment which requires specific instruments and optimized parameters (Banakar et al., 2020). The process of regeneration of protoplasts is highly necessary for the recovery of genome-edited plants through RNP-mediated genome editing. However, it could still be possible to evaluate the efficacy of CRISPR systems at cell level for a new plant species, having unoptimized protocol for the regeneration process. Alternative RNP transformation methods like *de novo* meristem induction could help bypass the regeneration process if any plant lacks robust methods of protoplast regeneration (Maher et al., 2020). For soybean, the protocol for regeneration of protoplast cells is available (Wei and Xu, 1988; Dhir et al., 1991). With the well-established method of direct delivery of engineered RNPs in this study, it would be feasible to breed novel traits of soybean and other related bean species without the use of any stable transformation methods.

## Conclusion

In summary, we describe here CRISPR/Cas9-based gene editing in soybean leaf protoplasts from young seedlings transformed with preassembled CRISPR-RNP mediated by PEG as a fast and low-cost approach to developing mutant lines for plant biology and biotechnology studies. Although the mutation efficiency was found to vary according to each sgRNA utilized at the gene (Glyma06g15080), the targeted deep and Sanger sequencing showed a range of mutational profiles (ranging from 4.2 to 18.1%) that resulted in frameshift types predicted to cause a premature stop codon at mRNA and cause loss of function in *GmCPR5* alleles. Combining confocal fluorescence microscopy to visualize viable cells and stained CRISPR cells is an important checkpoint to improve the targeted mutagenesis. Transformed protoplasts stained can also be subjected to fluorescence-activated cell sorting (FACS) providing enrichment mutants cells that could be used for cell embryogenic cultivation. This, despite a great challenge, presents potential in the face of increasingly future studies on regeneration. Finally, this platform used here as a proof of concept can also be used as a strategy to apply transient genes and study the function and regulation of the genes.

## Data Availability Statement

The original contributions presented in this study are publicly available. This data can be found here: NCBI, PRJNA785774.

## Author Contributions

SS initiated, designed, and conducted the analyses. CZ helped to initiate the work and co-designed the experiments. JN and AH contributed to the data preparation. RN and SA-T supervised the research. All authors contributed to the article and approved the submitted version.

## Conflict of Interest

The authors declare that the research was conducted in the absence of any commercial or financial relationships that could be construed as a potential conflict of interest.

## Publisher’s Note

All claims expressed in this article are solely those of the authors and do not necessarily represent those of their affiliated organizations, or those of the publisher, the editors and the reviewers. Any product that may be evaluated in this article, or claim that may be made by its manufacturer, is not guaranteed or endorsed by the publisher.

## Footnotes

1
https://www.soybase.org/

2
http://www.rgenome.net/cas-analyzer/

## Supplementary Material

Supplementary Figure 1 Agarose gel photographs of PCR amplicons during targeted deep and Sanger sequencing. **(A,B)** Gel images of first **(A)** and second **(B)** rounds of multiplex nested PCR products for targeted deep sequencing using primers listed in Supplementary Table 2. **(C)** Gel images of PCR amplicons for Sanger sequencing. Lanes M, DNA ladders; T1–T5, Protoplast transformants; C, untransformed wild type (control); T, transformed with RNPs. The expected size of PCR fragments is indicated with red arrows.

Supplementary File 1 Chromatogram files in ABI format from Sanger sequencing assay for the control and protoplast transformants of T2 and T4.

## Abbreviations

9M CPW: 9% mannitol with CPW salts
Aa: amino acids
CPR5: constitutive pathogen response 5
CPW: cell and protoplast washing
crRNA: CRISPR RNA
ESID: electronically switchable illumination and detection module
FACS: fluorescence-activated cell sorting
FDA: fluorescein diacetate
GFP: green fluorescent protein
GMO: genetically modified organism
gRNA: guide RNAs, InDels, insertions and deletions
NCBI: National Center for Biotechnology Information
NHEJ: non-homologs end joining
PEG: polyethylene glycol
RNP: ribonucleoproteins
T7E1: T7 endonuclease I
T-DNA: transfer DNA.
