# Supplementary figures and images for: A DNA-Free Editing Platform for Genetic Screens in Soybean via CRISPR/Cas9 Ribonucleoprotein Delivery
Source: Front Plant Sci. 2022 Jul 12;13:939997. doi: 10.3389/fpls.2022.939997 (PMC9315425; doi:10.3389/fpls.2022.939997)

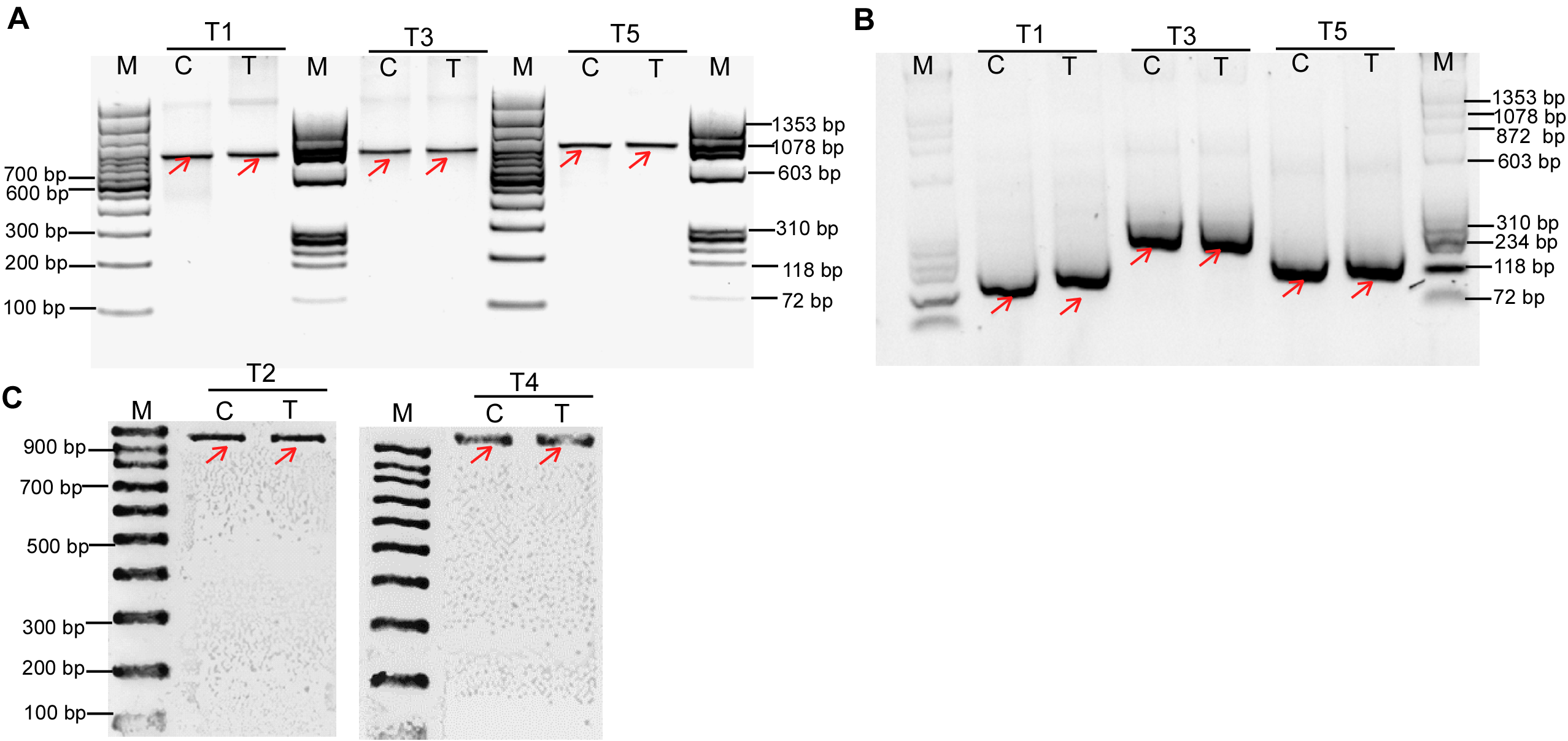

Supplement: Supplementary Figure 1 — Agarose gel photographs of PCR amplicons during targeted deep and Sanger sequencing. (A,B) Gel images of first (A) and second (B) rounds of multiplex nested PCR products for targeted deep sequencing using primers listed in Supplementary Table 2. (C) Gel images of PCR amplicons for Sanger sequencing. Lanes M, DNA ladders; T1–T5, Protoplast transformants; C, untransformed wild type (control); T, transformed with RNPs. The expected size of PCR fragments is indicated with red arrows. [file Image_1.tif]
