# Supplementary material for: A DNA-Free Editing Platform for Genetic Screens in Soybean via CRISPR/Cas9 Ribonucleoprotein Delivery
Source: Front Plant Sci. 2022 Jul 12;13:939997. doi: 10.3389/fpls.2022.939997 (PMC9315425; doi:10.3389/fpls.2022.939997)
Supplement: Supplementary file 3 [file Table_1.docx]

| **gRNA** | **Target** | **Sequence (5' to 3')** | **Position (bp)** | **Exons** | **Cleavage Position (%)** | **Strand orientation** | **GC Contents**  **(%, without PAM)** | **Out-of-frame Score** | **Mismatches** | | | | |
| --- | --- | --- | --- | --- | --- | --- | --- | --- | --- | --- | --- | --- | --- |
|  |  |  |  |  |  |  |  |  | **0** | **1** | **2** | **3** | **4** |
| 1 | T1 | AGGCTGCGGCGTTCAAACGA**CGG** | 146–168 | E1 | 5.7 | + | 60 | 84.2 | 1 | 0 | 0 | 1 | 3 |
| 2 | T2 | GGCGGCGAACAAGAACTCTA**GGG** | 171–193 | E1 | 6.1 | - | 55 | 59.1 | 0 | 0 | 0 | 0 | 11 |
| 3 | T3 | GTCTCCCAGTCATCTTTCGA**TGG** | 591–613 | E2 | 21.3 | + | 50 | 77.9 | 1 | 0 | 0 | 3 | 6 |
| 4 | T4 | GGTGATATTGTTACAGAGGT**CGG** | 1430 –1452 | E4 | 50.6 | + | 40 | 62.4 | 0 | 0 | 2 | 6 | 57 |
| 5 | T5 | AGCTTTAGTAATCCGCTCGT**AGG** | 2034 –2056 | E4 | 71.4 | - | 45 | 86.7 | 1 | 0 | 0 | 0 | 2 |

**Supplementary Table S1**. List of sgRNAs designed to target soybean *CPR5* locus. PAM motifs in sgRNA sequences are indicated by bold
