# Supplementary material for: A DNA-Free Editing Platform for Genetic Screens in Soybean via CRISPR/Cas9 Ribonucleoprotein Delivery
Source: Front Plant Sci. 2022 Jul 12;13:939997. doi: 10.3389/fpls.2022.939997 (PMC9315425; doi:10.3389/fpls.2022.939997)
Supplement: Supplementary file 4 [file Table_2.DOCX]

**Supplementary Table S2**. List of PCR primers used in this study

| **Target site** | **Invitro cleavage, T7E1 and Deep Target sequencing (1^st^ PCR)** | | | | **Deep Target sequencing (2^nd^ PCR) and Sanger sequencing** | | | |
| --- | --- | --- | --- | --- | --- | --- | --- | --- |
|  | **Primer Sequence (5'-3')** | **Annealing temperature**  **(Tm °C)** | **Annealing**  **Regions (bp)** | **Amplicon size**  **(bp)** | **Primer Sequence (5'-3')** | **Annealing temperature**  **(Tm °C)** | **Annealing**  **Regions (bp)** | **Amplicon size**  **(bp)** |
| T1 | **F:**CAAATACGGGAACTAGGCGG | 62 | 234-214^a^ | 657 | **F:**CGCAAAGTTCAGAAGCCTCC | 58 | 86–105 | 148 |
|  | **R:**AGCGTTAAAGAAACACGTGA | 61 | 403–423 |  | **R:**GCCAAGAGGAAGGCCAATAG | 56 | 214–234 |  |
| T2 | **F:**CTTCACTGAAATTGCGACCC | 59 | 2-22^a^ | 931 | **F:**CTTCACTGAAATTGCGACCC | 59 | -22^a^ | 931 |
|  | **R:**TTGTGGCCAAAATCAGGG | 60 | 892-909 |  | **R:**TTGTGGCCAAAATCAGGG | 60 | 892-909 |  |
| T3 | **F:**CGCAAAGTTCAGAAGCCTCC | 58 | 86–105 | 660 | **F:**TTGCTTGATATTGGGCATTAC | 55 | 508–528 | 237 |
|  | **R:**CCAAACATGCGCTTCATCAC | 60 | 726–745 |  | **R:**CCAAACATGCGCTTCATCAC | 57 | 726–745 |  |
| T4 | **F:**GCCAGTGTAAGACAGACATCAA | 56 | 1103-1124 | 953 | **F:**GCCAGTGTAAGACAGACATCAA | 56 | 1103-1124 | 953 |
|  | **R:**GCTTTAGTAATCCGCTCGTAGG | 56 | 2034-2055 |  | **R:**GCTTTAGTAATCCGCTCGTAGG | 56 | 2034-2055 |  |
| T5 | **F:**TCTCCTGAAGAGAGCAGGGA^b^ | 57 | 1601–1620 | 693 | **F:**ACGAGGCATGGTGAACTCAA | 57 | 1937–1956 | 148 |
|  | **R1:**AACTGCCAATTGAGCAAAGG^b^ | 56 | 2275–2294 |  | **R2:**TGCCTGGGTTGATGGTGTAC | 58 | 2067–2085 |  |
|  | **F:**TCTCCTGAAGAGAGCAGGGA^c^ | 57 | 1601–1620 | 486 | **-** |  | - | - |
|  | **R2:**TGCCTGGGTTGATGGTGTAC^c^ | 58 | 2067–2085 |  | **-** |  | - | - |

^a^ Numbers indicates the location of designed primers at the 5′ flanking promoter regions of *GmCPR5 locus* (Glyma06g15080).

^b^ Primers used for T7E1 assay, and deep target 1^st^ PCR for the T5 target site.

^C^ Primers used for invitro cleavage assay for the T5 target site.
